# Prolonged Capillary Refill Time as an Early Sign of Compromised Circulation in an Infant With Supraventricular Tachycardia: A Case Report

**DOI:** 10.7759/cureus.75913

**Published:** 2024-12-17

**Authors:** Kazumasa Zensho, Kojiro Mitsui, Kayo Ogino

**Affiliations:** 1 Pediatrics, Okayama University Hospital, Okayama, JPN; 2 Pediatrics, Kurashiki Medical Center, Okayama, JPN; 3 Pediatric Cardiology, Kurashiki Central Hospital, Okayama, JPN

**Keywords:** capillary refill time, emergency medicine training, infant, pediatric advanced life support, pediatrics

## Abstract

Capillary refill time (CRT) is a valuable clinical sign in pediatric assessment, particularly in evaluating circulatory status. We present a case of a one-month-old infant with supraventricular tachycardia (SVT), who demonstrated prolonged CRT, emphasizing the importance of this physical examination finding in the context of other signs of compromised circulation.

## Introduction

Capillary refill time (CRT) is defined as the time required for skin color to return after blanching. CRT assessment remains a cornerstone in pediatric circulatory evaluation, despite variations in measurement technique and interpretation [1]. This assessment is critical in neonates, where traditional vital signs can be difficult to obtain accurately. Early detection of circulatory compromise is essential to prevent adverse outcomes. In neonates with supraventricular tachycardia (SVT), several factors complicate the interpretation of CRT, including their higher resting heart rate, immature autonomic regulation, and variable perfusion patterns [2,3]. Several factors influence CRT measurement technique and interpretation, including compression pressure, duration, and site of assessment. Understanding these variations and applying a consistent methodology can improve clinical utility in pediatric emergency care [4]. Careful consideration of neonatal-specific factors, such as the effect of ambient temperature on peripheral perfusion, the impact of varying skin pigmentation on visual assessment, and age-related variations in normal values, may enhance its clinical value [5-7]. Recent meta-analyses have shown that prolonged CRT has a sensitivity of 34.6% (95% CI: 23.9-47.1%) and a specificity of 92.3% (95% CI: 88.6-94.8%) for predicting mortality in children, with a positive likelihood ratio of 4.49 (95% CI: 3.06-6.57). This indicates that children with prolonged CRT have a four-fold increased risk of death compared to children with normal CRT [8].

In the context of neonatal SVT, prolonged CRT serves as an early warning sign of compromised circulation, often preceding other clinical manifestations. This early detection is critical because neonatal SVT can rapidly progress to cardiovascular collapse, due to limited compensatory mechanisms and the challenges of early diagnosis posed by non-specific presenting symptoms [9].

There is a knowledge gap regarding CRT assessment, particularly in neonatal emergency medicine. This case report demonstrates how standardized CRT assessment can contribute to the early detection of circulatory compromise in neonatal SVT.

## Case presentation

A previously healthy one-month-old infant, born at term with no significant perinatal complications or family history of cardiac disease, presented with a deteriorating general condition and decreased feeding volume over the previous day. Pregnancy was uneventful, with appropriate prenatal care, and there was no family history of cardiac disease, arrhythmias, or sudden death. The infant had been exclusively breastfed, with appropriate weight gain and normal development until the onset of symptoms. The infant's deterioration was progressive, with initial subtle changes in feeding behavior progressing to markedly decreased oral intake over 24 hours. On examination, he was lethargic, with occasional weak crying and minimal limb movement. His vital signs revealed unmeasurable blood pressure and a respiratory rate of 32 breaths/minute (normal range: 30-60). The patient's body temperature was 36.2°C, indicating mild hypothermia. No cyanosis was observed.

Physical examination revealed a significantly prolonged CRT of six seconds, not only on the right sole but also on the chest (Video 1). Peripheral pulses were absent and weak in all extremities. There was no obvious murmur, hepatomegaly, peripheral edema, or other signs of heart failure. The prolonged CRT, combined with hypothermia and altered mental status, suggested significant circulatory compromise. Electrocardiography revealed a narrow QRS tachycardia at a rate of 253 beats per minute (normal range for age: 100-150), with absent P waves, consistent with SVT. Echocardiography demonstrated decreased left ventricular contraction, without structural abnormalities. Laboratory findings were consistent with the clinical picture of circulatory compromise: venous blood gas analysis revealed metabolic acidosis (pH, 7.323; pCO2, 37.6 mmHg; HCO3-, 19.0 mM), likely due to tissue hypoperfusion, as evidenced by the elevated lactate of 6.1 mM. The combination of hyponatremia (131 mEq/L) and hyperkalemia (5.9 mEq/L) further supported the presence of significant circulatory compromise and metabolic derangement.

**Video 1:** Capillary refill time assessment technique in a one-month-old infant This video demonstrates the standardized technique for assessing capillary refill time (CRT) in a one-month-old infant with supraventricular tachycardia. The assessment was performed on the right sole, with the limb elevated slightly above the heart level. The examiner applied moderate pressure (approximately 3-7 N, equivalent to lifting two smartphones) for five seconds, followed by sudden release. Care was taken to avoid excessive toe extension during the measurement, as this may artificially prolong the refill time. To ensure measurement accuracy, CRT was also confirmed at other sites, including the chest wall. The prolonged CRT of six seconds indicated compromised peripheral circulation. The video clearly shows the blanching of the skin during compression and the delayed return of color after release, which aided in the early recognition of circulatory compromise.

**Figure 1:**
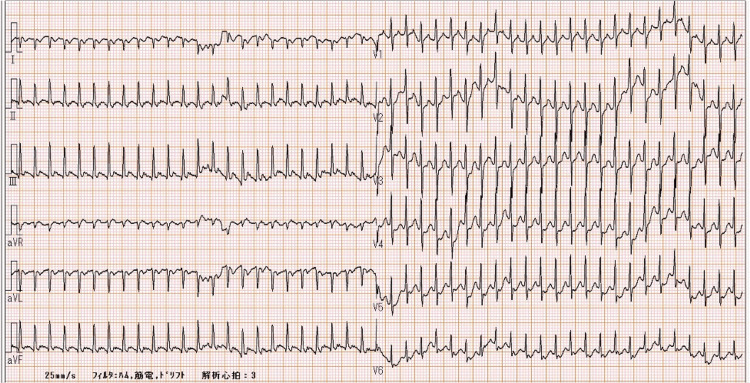
Twelve-lead electrocardiogram capturing the onset of supraventricular tachycardia A twelve-lead electrocardiogram demonstrates the transition from normal sinus rhythm to supraventricular tachycardia (SVT). The SVT shows a narrow QRS complex tachycardia at a rate of 253 beats per minute, with absent P waves. Note the abrupt onset of the tachyarrhythmia and the loss of clearly discernible P waves during the SVT episode, characteristic findings that help distinguish SVT from sinus tachycardia. The abrupt onset and regular narrow QRS complexes helped distinguish SVT from other tachyarrhythmias in infants. SVT typically shows a gradual rate change, while sinus tachycardia typically has P waves. The narrow QRS complexes and sawtooth waves distinguished this from atrial flutter, which has wider QRS complexes.

Given the presence of hemodynamic compromise and the patient's young age, adenosine was selected as the first-line treatment due to its rapid onset, short half-life, and established safety profile in neonates. A rapid bolus of adenosine (0.1 mg/kg) was given, which successfully terminated the tachycardia. After experiencing a second SVT episode one month later, oral flecainide was initiated, based on its efficacy in preventing recurrent SVT in infants and its favorable side effect profile compared to other antiarrhythmic medications. The patient was monitored with regular electrocardiograms. After six months of follow-up, no subsequent recurrences were observed (Video 2; Figure 1).

**Video 2:** Echocardiography showing decreased left ventricular systolic function during supraventricular tachycardia The left ventricular short-axis view on echocardiography revealed decreased contractility during supraventricular tachycardia (SVT). The video demonstrates reduced left ventricular wall motion, with an estimated ejection fraction of less than 40% during the SVT episode, indicating significant systolic dysfunction secondary to tachycardia-induced cardiomyopathy. Systolic dysfunction in SVT-induced cardiomyopathy is expected because the prolonged rapid heart rate results in increased myocardial oxygen demand and decreased coronary perfusion time, leading to a mismatch between myocardial oxygen supply and demand. Note the absence of structural cardiac abnormalities, pericardial effusion, or regional wall motion abnormalities.

## Discussion

This case highlights several important aspects of CRT assessment and interpretation in pediatric patients. The measurement technique requires careful standardization, with optimal compression strength equivalent to lifting two smartphones (3-7 N) and a recommended compression time of five seconds [10,11]. The use of a stopwatch and verbal timing is recommended for accuracy. Even in resource-limited settings, this standardized approach can be effectively implemented, as it requires minimal equipment and can be taught through simple demonstration and practice.

The laboratory findings in our case deserve special consideration in the context of the existing literature. Our patient's elevated lactate (6.1 mM) is consistent with previous findings showing that lactate levels >4.0 mM are associated with increased mortality in children with compromised circulation [12]. In addition, the observed hyponatremia (131 mEq/L) is a significant risk factor, as hyponatremia on admission has been reported to be independently associated with increased mortality and the need for mechanical circulatory support in children with impaired cardiac function [13].

The interpretation of CRT must consider multiple factors that can affect its accuracy. Room temperature, the patient's age, skin pigmentation, and body temperature all play crucial roles in proper interpretation [2,5-7]. As demonstrated in our case, the patient's mild hypothermia (36.2℃) may have potentially prolonged the CRT measurement. However, the presence of other signs of circulatory compromise and the magnitude of the CRT prolongation (greater than two seconds) suggested that the finding was pathological rather than merely temperature-related.

CRT assessment should be interpreted in conjunction with other bedside perfusion markers. While our patient had unmeasurable blood pressure, other important perfusion indicators include peripheral pulse quality (which, in our case, was weak and thready), skin mottling, peripheral-core temperature gradient, and mental status changes. Each of these markers has limitations: pulse quality assessment is subjective, skin mottling can be difficult to assess in darker skin tones, and temperature gradients require specialized equipment. In contrast, CRT provides a rapid, device-free assessment that can be performed in any setting.

In pediatric patients, a normal CRT is considered to be two seconds or less [8]. Prolonged CRT indicates compromised peripheral perfusion and should be interpreted alongside other clinical signs [1,14,15]. This measurement is particularly valuable within the Pediatric Advanced Life Support (PALS) algorithm, where it serves as an early indicator of shock and helps guide the timing and aggressiveness of interventions. In our case, the prolonged CRT, combined with altered mental status and unmeasurable blood pressure, triggered immediate therapeutic intervention according to the PALS protocol [9].

While automated devices for objective CRT measurement have been developed [16,17], these tools present both opportunities and challenges. Automated devices may offer improved accuracy and reduced interobserver variability compared with manual assessment. However, their cost, limited availability, and technical maintenance requirements make them impractical for widespread implementation, particularly in resource-limited settings. Therefore, standardized manual assessment techniques remain critical and should be emphasized in clinical training.

CRT is useful for diagnosis, monitoring treatment response, and guiding ongoing care. In our patient, normalization of CRT after adenosine administration provided immediate feedback on treatment efficacy. During follow-up, regular CRT assessment helped monitor for early signs of SVT recurrence and guide decisions about antiarrhythmic therapy. This highlights the value of incorporating CRT measurement into routine follow-up protocols for conditions associated with impaired circulation.

The integration of CRT assessment with PALS algorithms enhances clinical decision-making [15]. In particular, our case demonstrates how CRT can help identify cardiovascular compromise in infants with SVT, where traditional vital signs, such as blood pressure, may be difficult to obtain accurately. The role of CRT becomes even more critical in settings where advanced monitoring equipment is not readily available, making it an invaluable tool for early detection of circulatory compromise. This case supports the incorporation of CRT assessment into standard clinical protocols, which recommend CRT as a key component of circulatory assessment [9,18].

## Conclusions

While CRT assessment techniques continue to evolve and standardization remains incomplete, proper evaluation remains a valuable tool in pediatric circulatory assessment. This case study contributes to our understanding of CRT in pediatric emergency care. It shows how prolonged CRT can indicate SVT in infants, potentially allowing earlier recognition and intervention. Incorporating CRT assessment into neonatal assessment may improve early detection of circulatory compromise, particularly in conditions such as SVT. This case demonstrates how prolonged CRT, when interpreted alongside other clinical findings, can support the recognition of circulatory compromise in infants. Further research is needed on standardized CRT measurement, training for healthcare providers, and the monitoring of treatment response. This case extends CRT's established clinical utility by demonstrating its specific value in the early recognition and monitoring of neonatal SVT, where timely diagnosis remains challenging, despite its critical importance.
